# Cherry Valley Ducks Mitochondrial Antiviral-Signaling Protein-Mediated Signaling Pathway and Antiviral Activity Research

**DOI:** 10.3389/fimmu.2016.00377

**Published:** 2016-09-21

**Authors:** Ning Li, Tianqi Hong, Rong Li, Yao Wang, Mengjiao Guo, Zongxi Cao, Yumei Cai, Sidang Liu, Tongjie Chai, Liangmeng Wei

**Affiliations:** ^1^Sino-German Cooperative Research Centre for Zoonosis of Animal Origin of Shandong Province, College of Veterinary Medicine, Shandong Agricultural University, Tai’an City, China; ^2^Collaborative Innovation Centre for the Origin and Control of Emerging Infectious Diseases of Taishan Medical College, Tai’an City, China; ^3^Hainan Provincial Key Laboratory of Tropical Animal Reproduction and Breeding and Veterinary Medicine, Institute of Animal Husbandry and Veterinary Medicine, Hainan Academy of Agricultural Sciences, Haikou, China

**Keywords:** cherry valley duck, MAVS, molecular cloning, signaling pathway, antiviral ability

## Abstract

Mitochondrial antiviral-signaling protein (MAVS), an adaptor protein of retinoic acid-inducible gene I (RIG-I)-like receptors (RLRs)-mediated signal pathway, is involved in innate immunity. In this study, Cherry Valley duck MAVS (duMAVS) was cloned from the spleen and analyzed. duMAVS was determined to have a caspase activation and recruitment domain at N-terminal, followed by a proline-rich domain and a transmembrane domain at C-terminal. Quantitative real-time PCR indicated that duMAVS was expressed in all tissues tested across a broad expression spectrum. The expression of duMAVS was significantly upregulated after infection with duck Tembusu virus (DTMUV). Overexpression of duMAVS could drive the activation of interferon (IFN)-β, nuclear factor-κB, interferon regulatory factor 7, and many downstream factors (such as Mx, PKR, OAS, and IL-8) in duck embryo fibroblast cells. What is more, RNA interference further confirmed that duMAVS was an important adaptor for IFN-β activation. The antiviral assay showed that duMAVS could suppress the various viral replications (DTMUV, novel reovirus, and duck plague virus) at early stages of infection. Overall, these results showed that the main signal pathway mediated by duMAVS and it had a broad-spectrum antiviral ability. This research will be helpful to better understanding the innate immune system of ducks.

## Introduction

The innate immune response occurs in the early days of infection and functions as the first line of host defense against microbial pathogens. The pattern recognition receptors (PRRs) play a central role in detecting the pathogen-associated molecular patterns (PAMPs). The recognition of PAMPs initiates the innate immune response to microorganism, which is characterized by the induction of interferons (IFNs), IFN-stimulated genes (ISGs), and proinflammatory cytokines (1, 2).

PRRs comprise multiple family members, including toll-like receptors (TLRs), retinoic acid-inducible gene I (RIG-I)-like receptors (RLRs), and NOD-like receptors (NLRs). To date, RIG-I, melanoma differentiation factor 5 (MDA5), and Laboratory of genetics and physiology 2 (LGP2) constitute the RLRs family. RIG-I possesses two N-terminal caspase activation and recruitment domains (CARDs), these domains send the signals to downstream signaling molecules, followed by a central RNA helicase domain and a transmembrane (TM) domain at C-terminal, which are responsible for binding viral RNA (3). The structure of MDA5 is similar to that of RIG-I, but LGP2 lacks the CARDs. Although RIG-I and MDA5 harbor the similar structures and both can detect the viral double-stranded RNA (dsRNA) in the cytoplasm, the specific ligands recognized by the two PRRs are different. RIG-I senses short and 5′ triphosphate dsRNA, as well as the short synthetic dsRNA analog poly(I:C) (<1 kbp), MDA5 which can be activated by the long dsRNA and poly(I:C) (>1 kbp) (4). Upon activation of RIG-I and MDA5, CARD will recruit an adaptor protein that is located in the mitochondria to phosphorylate transcription molecular nuclear factor (NF)-κB and IFN regulatory factor 3/7 (IRF3/7), and induce the expression of cytokines, ultimately resulting in the establishment of innate immune response and the development of adaptive immunity (3).

Mitochondrial antiviral-signaling protein (MAVS), an adaptor protein in the RLRs signaling pathways, has been identified recently (5). It may interact with CARDs of RIG-I and MDA5 via its N-terminal CARD domain. Overexpression of MAVS increased the expression of IFN-α/β and ISGs through activation of IRF3/7 and NF-κB; moreover, the induction of type I IFNs and antiviral immune response to viral infection were impaired after knockdown of MAVS. These results indicate that the MAVS is essential for innate immune response (5, 6). Since this adaptor protein was simultaneously identified and functionally characterized by other research groups, it has also been designated, VISA, MAVS, or Cardif (5, 7, 8). In recent years, MAVS has been cloned and reported in many species, including monkeys (9), cat (10), mice (11), pigs (12), chickens (13), and some fish (14–16).

Cherry Valley ducks are widely reared and create huge economic benefits in China. Over the past few years, however, outbreaks of many diseases, such as avian influenza, novel duck reovirus (NDRV), and duck Tembusu virus (DTMUV) disease (17–19), have brought great changes to the duck industry and caused major economic losses. Therefore, further studies of the innate immune response of ducks will be important for controlling acute infectious diseases. In the current study, Cherry Valley duck MAVS (duMAVS) was cloned and its tissue distribution in healthy ducks was examined. Additionally, the expression profiles of duMAVS were examined at the early stages of viral infection. The main signaling pathway and antiviral activity of duMAVS were also investigated. These results will be helpful in further understanding the role of duMAVS in the duck immune system.

## Materials and Methods

### Virus, Cell, and Animals

DTMUV-FX2010 strain, NDRV, and duck plague virus (DPV)-GM strain were used in this study. They belong to Flaviviridae, Reoviridae, and Herpesviridae, respectively. The three viruses were all isolated from the clinical infected ducks and virus stocks were propagated in duck embryo fibroblasts (DEFs) (20–22). Viral titers were determined as median tissue culture infective dose (TCID_50_)/mL by infection of DEFs and calculation by Reed and Muench method, respectively (23).

DEFs, derived from the 11-day-old duck embryo, were cultured in Dulbecco’s modified Eagle medium (GIBCO, Grand Island, NY, USA) supplemented with 10% fetal bovine serum (Transgen, Beijing, China) at 37°C in an atmosphere with 5% (v/v) CO_2_.

One-day-old Cherry Valley ducks were purchased from a farm, and raised in isolation for the experiment until 3 weeks old. Several common diseases in ducks were detected in this study to determine the baseline status of the healthy ducks. Specifically, when the ducks were 20 days old, hemagglutination inhibition test was used to detect the antibodies of avian influenza and Newcastle disease, respectively. Additionally, the enzyme-linked immunosorbent assays were performed to determine the negative for NDRV, DTMUV, and DPV, respectively (24). And all ducks were confirmed to be negative for the above viruses.

### Cloning and Bioinformatic Analysis of the duMAVS

Total RNA was extracted from healthy duck spleen using TRIzol reagent (Takara, Dalian, China) and 1 μg total RNA was reverse-transcribed with HiScript^R^II One Step RT-PCR kit (Vazyme, Nanjing, China). To obtain partial sequence of duMAVS, degenerate primers (duMAVS-F1, duMAVS-R1, duMAVS-F2, and duMAVS-R2) were designed based on the predicated gene in the Genbank (XM_013102297.1) (Table 1). Moreover, other primers, duMAVS-F3 and duMAVS-R3 (Table 1), were designed for cloning the 5′ sequence according to the multiple alignments of the reported MAVS sequence: chicken (*Gallus gallus*; NM_001012893.1), goose (*Anser cygnoides* domesticus; XM_013182243.1), and duck (*Anas platyrhynchos*; NM_001310828.1). The products of primers were fused using fusion PCR. Eventually, the coding region gene of MAVS was ligated into the pMD18-T vector (Takara, Dalian, China) and sequenced.

**Table 1:** **Primer sequences used in this study**.

Primer name	Primer sequence (5′-3′)	Purpose
duMAVS-F1	ACATCCTGAGGAACATGGAC	Gene cloning
duMAVS-R1	TGCAGCCGGGCGTACACCAG	
duMAVS-F2	CGGCTGCAGAAATAGAGGAG	Gene cloning
duMAVS-R2	TAATTGGGTTTGGGGTTTGA	
duMAVS-F3	CGAGCCAGGATGGGCTT	Gene cloning
duMAVS-R3	ATCGACCAGCGACGCCACGT	
duMAVS-CARD-F	ATGGGCTTCGCGGAGGACAA	Gene cloning
duMAVS-CARD-R	GTAGACCTGCTGCAGCTCTTC	
duMAVS-dCARD-F	GACCTCTACCAAACCCCTCC	Gene cloning
duMAVS-dCARD-R	CTATTTCTGCAGCCGGGCGT	
duMDA5-CARD-F	ATGTCGACGGAGTGCCGAGA	Gene cloning
duMDA5-CARD-R	ATTTCCACTTAAATCATCTG	
qduMAVS-F	ACATCCTGAGGAACATGGAC	RT-qPCR
qduMAVS-R	AGACCTCCTGCAGCTCTTCG	
IFN-α-F	TCCTCCAACACCTCTTCGAC	RT-qPCR
IFN-α-R	GGGCTGTAGGTGTGGTTCTG	
IFN-β-F	AGATGGCTCCCAGCTCTACA	RT-qPCR
IFN-β-R	AGTGGTTGAGCTGGTTGAGG	
IL-1β-F	TCATCTTCTACCGCCTGGAC	RT-qPCR
IL-1β-R	GTAGGTGGCGATGTTGACCT	
IL-2-F	GCCAAGAGCTGACCAACTTC	RT-qPCR
IL-2-R	ATCGCCCACACTAAGAGCAT	
IL-6-F	TTCGACGAGGAGAAATGCTT	RT-qPCR
IL-6-R	CCTTATCGTCGTTGCCAGAT	
IL-8-F	AAGTTCATCCACCCTAAATC	RT-qPCR
IL-8-R	GCATCAGAATTGAGCTGAGC	
Mx-F	TGCTGTCCTTCATGACTTCG	RT-qPCR
Mx-R	GCTTTGCTGAGCCGATTAAC	
OAS-F	TCTTCCTCAGCTGCTTCTCC	RT-qPCR
OAS-R	ACTTCGATGGACTCGCTGTT	
PKR-F	AATTCCTTGCCTTTTCATTCAA	RT-qPCR
PKR-R	TTTGTTTTGTGCCATATCTTGG	

*Quantitative RT-PCR analysis of duMAVS in healthy and viral infected ducks*.

A phylogenic tree was made using the MEGA 5.0 with the neighbor-joining method. BLAST program was used to analyze sequence homology in the nucleotide database of the National Center for Biotechnology Information website (NCBI).^1^ Amino acid sequences were aligned by Clustalx and edited by BOXSHADE.^2^ The functional domains of duMAVS were predicated by the Sample Modular Architecture Research Toll (SMART).^3^

Five healthy ducks were randomly selected and tissues were collected for tissue distribution analyses of duMAVS, including heart, liver, spleen, lung, kidney, brain, cerebellum, brainstem, thymus, pancreas, bursa of Fabricius, trachea, esophagus, muscular stomach, glandular stomach, skin, muscle, duodenum, jejunum, ileum, and cecum. The remaining ducks were randomly divided into two groups. In group A, ducks were intramuscularly injected with DTMUV (0.4 mL per duck). In group B, all ducks were inoculated with 0.4 mL sterile phosphate buffer solution in the same manner as a negative control group. Five ducks from each group were killed at 1, 3, and 5 days post infection (dpi), and the spleen and brain were collected for RNA extraction. The extraction and reverse transcription of total RNA were performed as described above. The primers of qduMAVS-F and qduMAVS-R were selected for Quantitative real time PCR (RT-qPCR) (Table 1). RT-qPCR was conducted with the 7500 Fast Real-Time PCR System (Applied Biosystems, CA, USA) using the SYBR Green PCR kit (Vazyme, Nanjing, China). RT-qPCRs consisted of 20 μL volume and conditions as follows: one cycle at 95°C for 5 min, 40 cycles of denaturation at 95°C for 10 s and extension at 60°C for 34 s, followed by a dissociation curve analysis. Each sample was analyzed in triplicate.

The remaining ducks were observed for clinical symptoms for 9 days. All operations were carried out in accordance with the guidelines of the Committee on the Ethics of Animals of Shandong Agricultural University (NO. SDAUA-2015-004).

### Construction of Expression Plasmids

The DNA fragment containing the open reading frame (ORF) of duMAVS was subcloned into the pCDNA3.1(+)-HA-His plasmid, leading to the pC-duMAVS-HA expression construct. The duMAVS-CARD (only with CARD-like domain), duMAVS-dCARD (without CARD-like domain), and duMDA5-CARD (only with MDA5-CARD) were amplified by PCR with the primers in Table 1. All fragments were subcloned into pCDNA3.1(+)-HA-His by Hieff Clone™ Multi One Step Cloning Kit (Yeasen, Shanghai, China), called the pC-duMAVS-CARD-HA, pC-duMAVS-dCARD-HA, and pC-duMDA5-CARD-HA, respectively. All recombinant expression plasmids were confirmed by sequencing.

### Transfection and Luciferase Assays

DEFs were cultured overnight in 24-well plates at 37°C before transfection. The luciferase reporter plasmids (pGL3-IFN-β-Luc and pGL3-IRF7-Luc) used were derived from chickens as previously described (25). The commercialization pGL3-NF-κB plasmid was purchased from Agilent (Santa Clara, CA, USA). The pRL-TK plasmid (Promega, Madison, WI, USA) acted as an internal control to normalize transfection efficiency and poly I:C was purchased from Sigma (Sigma-Aldrich Corp., St. Louis, MO, USA). Various expression plasmids (pC-duMAVS-HA, pC-duMAVS-CARD-HA, and pC-duMAVS-dCARD-HA), empty vector and poly I:C (500 ng/well) with reporter plasmid (100 ng/well), and pRL-TK plasmid (50 ng/well) were transiently cotransfected into 80% confluent DEFs by Lipofectamine 2000 (Invitrogen, Carlsbad, CA, USA) following the instructions. The cells were lysed and harvested, luciferase activities were detected with a dual-luciferase reporter assays system (Beyotime, Wuhan, China) at 24 h post-transfection (hpt) according to the manufacturer’s instructions. All luciferase reporter assays were repeated three times.

### Cytokines Induced by duMAVS Overexpression in DEFs

DEFs were seeded in 6-well plates and cultured overnight at 37°C. Expression plasmid pC-duMAVS-HA, empty vector and poly I:C (2 μg/well) were transfected into DEFs in 200 μL of Opti-MEM using Lipofectamine 2000 (6 μL) (Invitrogen, Carlsbad, CA, USA), and then cultured at 37°C. After 24 hpt, cells were harvested and the expression of cytokines induced by duMAVS was detected by RT-qPCR method as described above (Table 1).

### Antiviral Activity of duMAVS

For antiviral assay, DEFs were transfected with duMAVS full length in 6-well plates as described above. After 24 hpt, DEFs were infected with DTMUV, NDRV, and DPV, respectively. Cells infected with viruses were washed with PBS after 1 h of adsorption. At 12, 24, and 36 h post infection (hpi), samples were collected for RNA and DNA extraction. The extraction and reverse transcription of total RNA were performed as described above. The extraction of DNA was performed by the conventional method. In brief, 12.5 μL proteinase K and 50 μL 10% SDS were added into the samples (437.5 μL) for 30 min of water bath to dissolve samples and denature protein. Subsequently, phenol, chloroform, and other organic solvent were used to extract and purify the DNA, and each sample 15 μL of water was added at the end. Viral titers were determined by RT-qPCR methods (20, 26, 27).

### RNA Interference

siRNA sequences (psiMAVS1, psiMAVS2, and psiMAVS3) targeting the different positions of duMAVS were prepared. siMAVS and negative control siRNA were synthesized by GenePharma (GenePharma, Shanghai, China). DEFs were transfected with the siRNAs with Lipofectamine 2000 reagent (Invitrogen, Carlsbad, CA, USA). The efficiencies of the siRNAs were measured by RT-qPCR.

### Statistical Analysis

The relative expression levels for detected genes were determined using the duck β-actin gene as the internal control and were calculated using the 2^−ΔΔCt^ method. All data were expressed as means ± SDs, and statistical analyses were performed by GraphPad Prism 5 software (GraphPad Software Inc., San Diego, CA, USA). Student’s *t* test was performed to evaluate the differences. The significant and highly significant differences were set as *P* < 0.05 and *P* < 0.01, respectively.

## Results

### Molecular Cloning and Sequence Analysis of duMAVS

**Figure 1:**
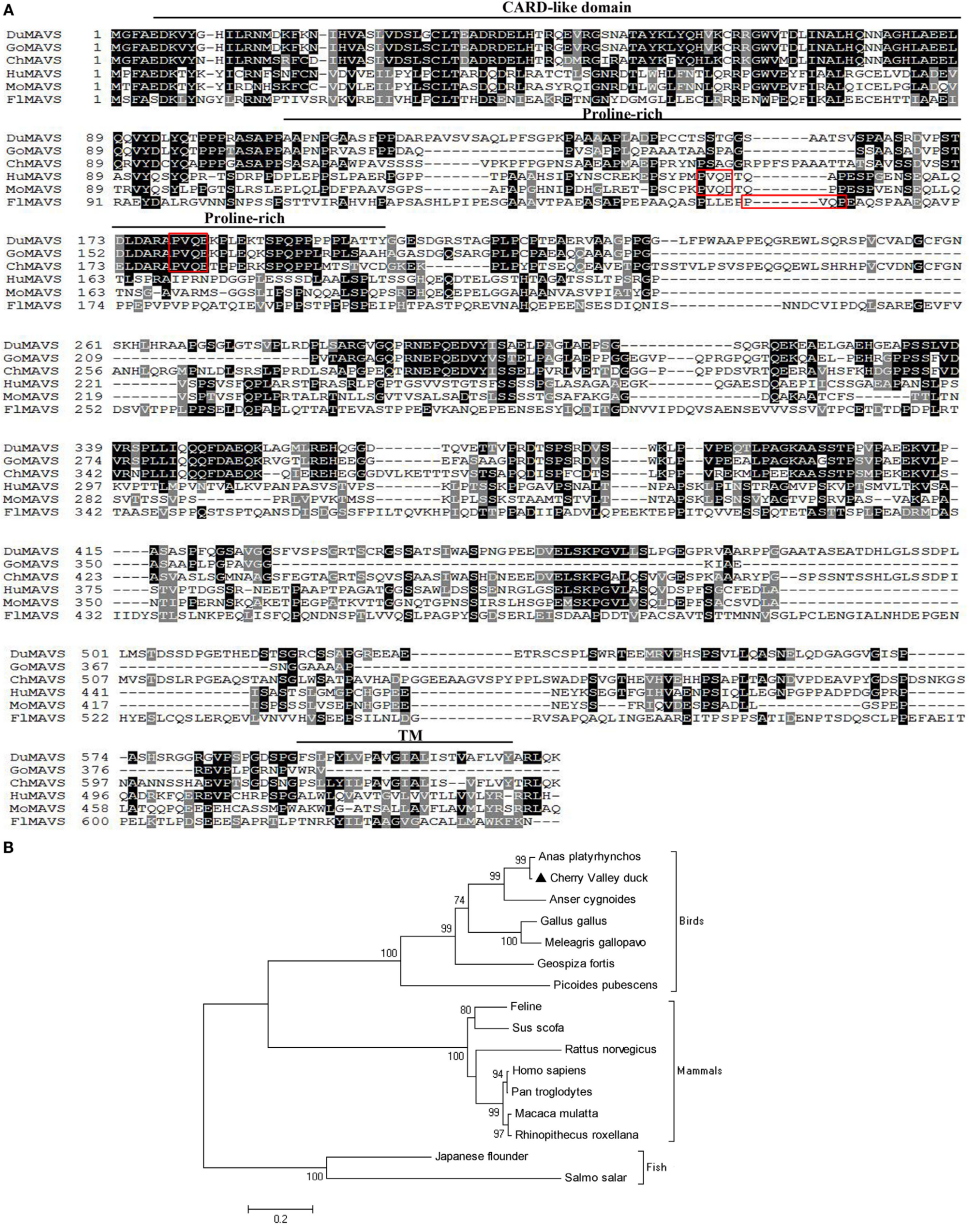
**Characterization of duMAVS**. **(A)** amino acid alignment of duck (KX290106), goose (XM_013182243.1), chicken (NM_001012893.1), human (DQ174270.1), mouse (DQ174271.1), and Japanese flounder (HM070252.1) MAVS. Alignment was performed using Clustal X program and edited with Boxshade. The MAVS sequences were shown for Cherry Valley ducks (Du), goose (Go), chicken (Ch), human (Hu), mouse (Mo), and Japanese flounder (Fl). Black shading indicated amino acid identity; gray shading indicated similarity (50% threshold). The CARD-like domain, proline-rich domain and TM were indicated in this figure. The red box indicated the TRAF2 binding motif. **(B)** A Phylogenic tree based on MAVS between Cherry Valley ducks and other species. Neighbor-joining tree was generated using MEGA 5.0 and a 1,000 – bootstrap analysis was performed. The scale bar was 0.2. GenBank accession nos.: *Anas platyrhynchos* MAVS, KJ466052.1; Cherry Valley duck MAVS, KX290106; *Anser cygnoides* MAVS, XM_013182243.1; *Gallus gallus* MAVS, NM_001012893.1; *Meleagris gallopavo* MAVS, XM_010710653.1; *Geospiza fortis* MAVS, XM_005427752.1; *Picoides pubescens* MAVS, XM_009907829.1; Feline MAVS, KT375569.1; *Sus scrofa* MAVS, EU082069.1; *Rattus norvegicus* MAVS, NM_001005556.1; Homo sapiens MAVS, DQ174270.1; Pan troglodytes MAVS, KC415006.1; *Macaca mulatta* MAVS, DQ842019.1; *Rhinopithecus roxellana* MAVS, XM_010378951.1; Japanese flounder MAVS, HM070252.1; *Salmo salar* MAVS, FJ854361.1.

The coding sequence of duMAVS was obtained from Cherry Valley duck spleen using the primers (duMAVS-F1/R1, duMAVS-F2/R2, and duMAVS-F3/R3). As shown in Figure 1A, the full length cDNA consists of a 1,860 bp ORF that encodes 619 amino acids, and its sequence has been deposited in GenBank (KX290106). The protein domains were predicated by SMART program and the results indicated that duMAVS contained three characteristic domains: a CARD-like domain at its N-terminus, a central proline-rich domain and a hydrophobic TM domain at its C-terminus (Figure 1A). Multiple alignment analysis of duMAVS revealed that duMAVS had the PVQE sequence, a TRAF2 binding motif, at 179–182 amino acid (Figure 1A), which is also consistent with human and other vertebrates MAVS (8, 15).

Alignment analysis of the deduced amino acid sequence of duMAVS showed that although the full length duMAVS only exhibited 17.4–77.7% identities to other species, the CARD had identities of 25.0% to flounder, 34.1% to mouse, 35.2% to human, and 80.7% to chicken (Table 2). As was expected, duMAVS had a higher identity to the goose (94.3%) (Table 2). A phylogenetic tree was generated based on the full length of MAVS, and the results showed that there were three major branches (Figure 1B). duMAVS and other birds MAVSs were in the same subgroup, which is confirmed by the high bootstrap values. Meanwhile, mammal and fish MAVSs were in the specific branches. These results demonstrated duMAVS was closely related to the MAVS of other birds, and the genetic relationship between duMAVS and goose was higher than those of the relationship to other.

**Table 2:** **Homology analysis of MAVS protein between Cherry Valley ducks and other species (% amino acid sequence identities^a^)**.

Species	Cherry Valley duck MAVS	
	Full length	CARD (amino acid 5–92)
Goose	77.7	94.3
Chicken	58.4	80.7
Human	22.0	35.2
Mouse	20.0	34.1
Flounder	17.4	25.0

*^a^Percent amino acid sequence homology was calculated by MegAlign software with Clustal W method. GenBank accession numbers were in Figure 1 legend*.

### Tissues Distribution of duMAVS

**Figure 2:**
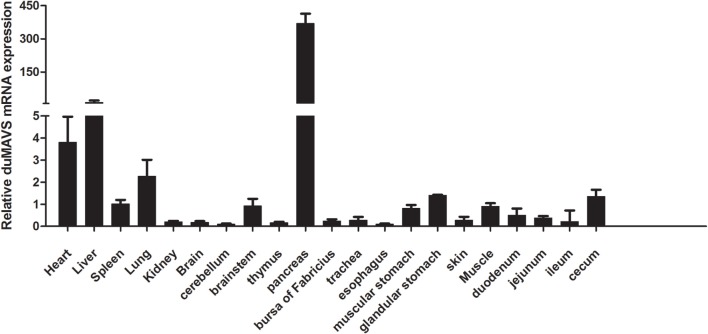
**The result of tissue distribution of duMAVS transcripts in the healthy Cherry Valley ducks**. The relative mRNA levels were normalized to the expression of the β-actin gene from various tissues. The data were normalized to the spleen and error bars indicated the SD.

To investigate the tissues distribution of duMAVS, total RNA was extracted separately from heart, liver, spleen, lung, kidney, brain, cerebellum, brainstem, thymus, pancreas, bursa of Fabricius, trachea, esophagus, muscular stomach, glandular stomach, skin, muscle, duodenum, jejunum, ileum, and cecum. RT-qPCR was performed to analyze the expression levels of duMAVS mRNA in the healthy Cherry Valley ducks. As shown in Figure 2, duMAVS was expressed in all tested tissues, strongly in heart, liver, and lung, with the highest in the pancreas. However, lower expressions were observed in spleen, kidney, brain, thymus, bursa of Fabricius, muscular stomach, glandular stomach, and cecum, etc. The broad expression profiles of duMAVS across the different tissues suggest that the role of duMAVS might have a certain universality in innate immunity.

### Expression Profiles of duMAVS in the Viral Infected Ducks

**Figure 3:**
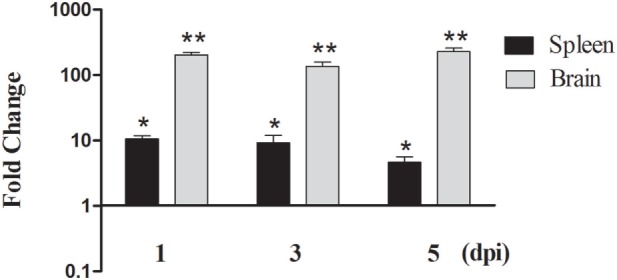
**Analysis of duMAVS transcript in ducks at early stages of DTMUV infection**. The fold change was calculated by the experimental ducks versus control ducks at the same time point, using the 2^−ΔΔCt^ method. All data were expressed as means ± SD (*n* = 5), and Student’s *t* test was performed to evaluate the differences. *Significant difference (*P* < 0.05); **highly significant difference (*P* < 0.01).

duMAVS transcript was detected in the spleen and brain during infection with DTMUV. As shown in Figure 3, the expression of duMAVS was upregulated at 1, 3, and 5 dpi after infection with DTMUV. In the spleen, the duMAVS expression was increased to 10.56-fold compared to the uninfected ducks at 1 dpi, and followed by 9.19- and 4.63-fold at 3 dpi and 5 dpi, respectively (*P* < 0.05). Meanwhile, the duMAVS mRNAs were significantly upregulated 203.90-fold at 1 dpi in the brain, and peaked at 5 dpi (227.83-fold, *P* < 0.01). The results indicated that duMAVS was involved in the host immune response to DTMUV infection.

### Functional Characteristic of duMAVS

**Figure 4:**
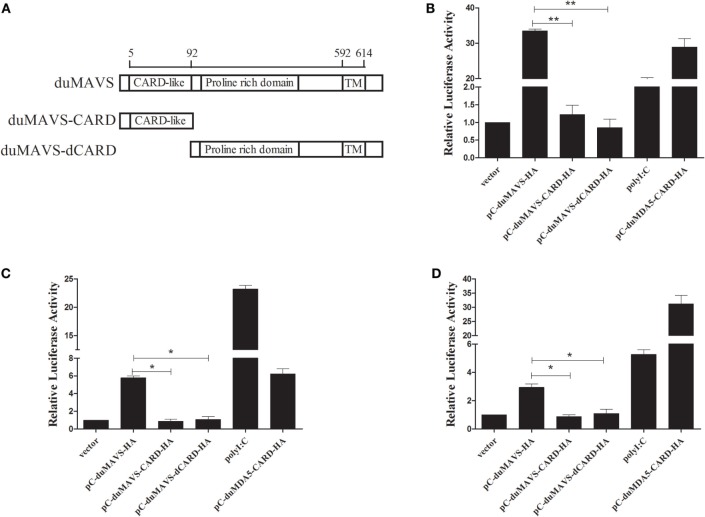
**The results of IFN-β, NF-κB, and IRF7 dual-luciferase reporter gene assay**. **(A)** Schema graph of the different duMAVS mutant plasmids used in this study. Various expressing plasmids (500 ng/well) were co-transfected with 100 ng/well reporter plasmids, **(B)** pGL3-IFN-β, **(C)** pGL3-NF-κB, and **(D)** pGL3-IRF7 with 50 ng/well pRL-TK plasmid. Cells transfected with polyI:C (500 ng/well) and pC-duMDA5-CARD-HA (500 ng/well) were used as the positive control group. After 24 h, cells were harvested and luciferase activity was measured. Data were means from three independent experiments and each experiment was analyzed in triplicate. Student’s *t* test was performed to evaluate the differences. *Significant difference (*P* < 0.05); **highly significant difference (*P* < 0.01).

MAVS acts as an important adaptor protein in RLRs-mediated signaling pathway, and it has been demonstrated that MAVS can activate IFN-α/β expression in mammals and fish (5, 12, 28). In order to investigate whether duMAVS can activate type I IFNs expression, and to clarify which part of duMAVS plays a major role in this process, mutant plasmids were constructed (Figure 4A). Various expressing plasmid and reporter plasmids were co-transfected into DEFs for luciferase reporter assay. As shown in Figure 4B, duMAVS could significantly activate IFN-β luciferase activities compared to empty vector (33.54-fold, *P* < 0.05), but the data demonstrated that duMAVS-CARD and dCARD mutants lost the ability to induce the expression of IFN-β, and there was a significant difference compared to duMAVS (Figure 4B).

Transcription factors, such as NF-κB and IRF3/7, coordinately regulate the expression of type I IFNs. To explore whether the duMAVS was involved in the activation of NF-κB and IRF7, the duMAVS expression plasmid was cotransfected with the luciferase reporter plasmids, pGL3-NF-κB-Luc and pGL3-IRF7-Luc. It was found that duMAVS could drive NF-κB and IRF7 activation (5.80- and 2.93-fold, respectively; Figures 4C,D). However, the duMAVS-CARD and dCARD mutants were unable to activate NF-κB and IRF7 (Figures 4C,D). These results of luciferase reporter assay proved that duMAVS overexpression could induce IFN expression by activating NF-κB and IRF7, but its CARD and dCARD domains were required for this induction.

### Cytokines and ISGs Expression Induced by duMAVS Overexpression in DEFs

**Figure 5:**
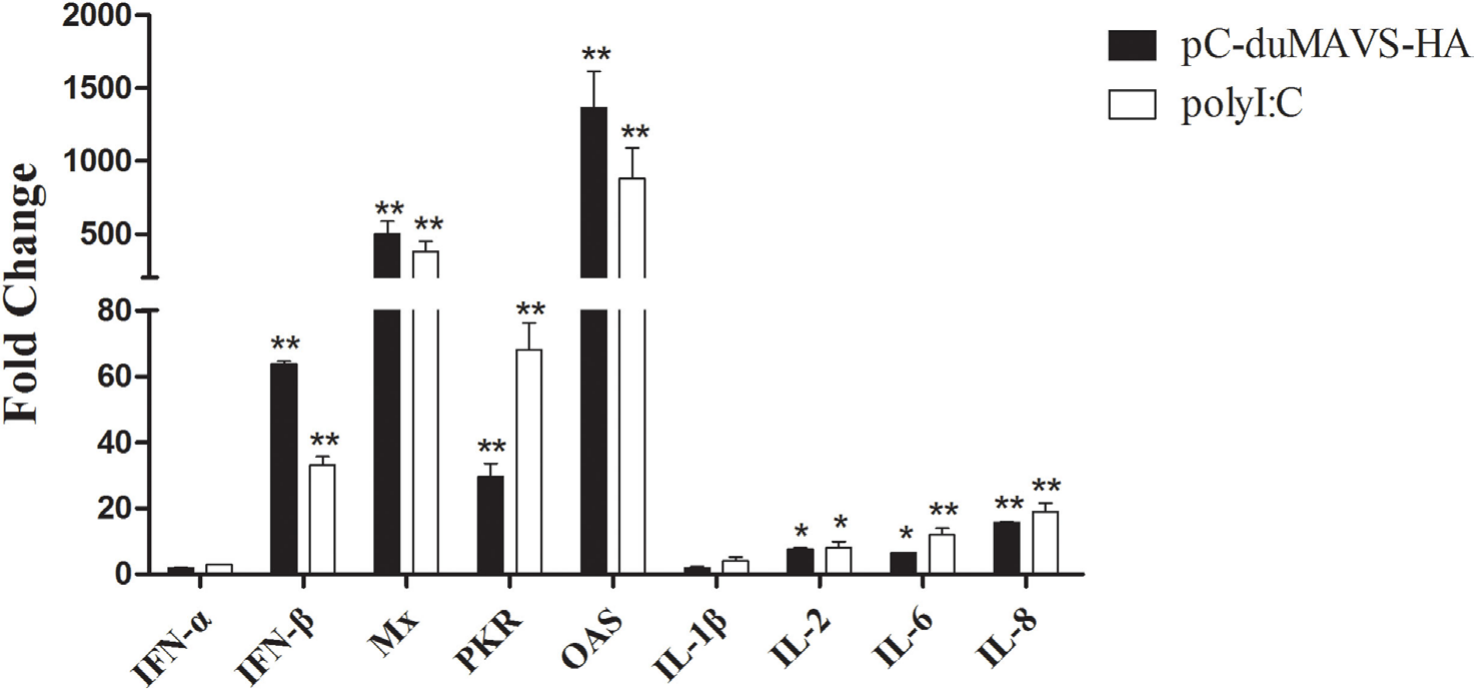
**The expression of downstream factors induced by duMAVS overexpression in DEFs**. The empty vector and pC-duMAVS-HA were transfected into DEFs. Cells transfected with polyI:C (2 μg/well) were used as the positive control. The cells were collected at 24 hpt, analyzing the inducible genes expression by RT-qPCR. The relative expression levels were normalized to the β-actin gene and calculated by the 2^−ΔΔCt^ method. The fold change was calculated by the experimental groups versus control groups at the same time point. Data were means from three independent experiments and each experiment was analyzed in triplicate. Student’s *t* test was performed to evaluate the differences. *Significant difference (*P* < 0.05); **highly significant difference (*P* < 0.01).

To identify what cytokines and ISGs could be induced by duMAVS, the expressions of IFN-α, IFN-β, Mx, PKR, OAS, IL-1-β, IL-2, IL-6, and IL-8 were analyzed using RT-qPCR method. After the overexpression of duMAVS in DEFs, the transcripts levels of type I IFNs were elevated and IFN-β expression upregulated 63.85-fold (*P* < 0.01, Figure 5). Notably, the expressions of ISGs, including Mx, PKR, and OAS significantly upregulated, and among them the Mx and OAS were increased more than 500- and 1,000-fold, respectively (*P* < 0.01, Figure 5). By contrast, the expressions of proinflammatory cytokines, such as IL-1β, IL-2, IL-6, and IL-8, were increased to different degrees after duMAVS transfection with the greatest increase of IL-8 (15.54-fold, *P* < 0.01; Figure 5). These results indicated overexpression of duMAVS full-length could significantly upregulate IFN-β and many ISGs, especially Mx and OAS. Furthermore, duMAVS overexpression was capable of elevating the proinflammatory cytokine induction to a certain extent.

### Antiviral Activity of duMAVS

**Figure 6:**
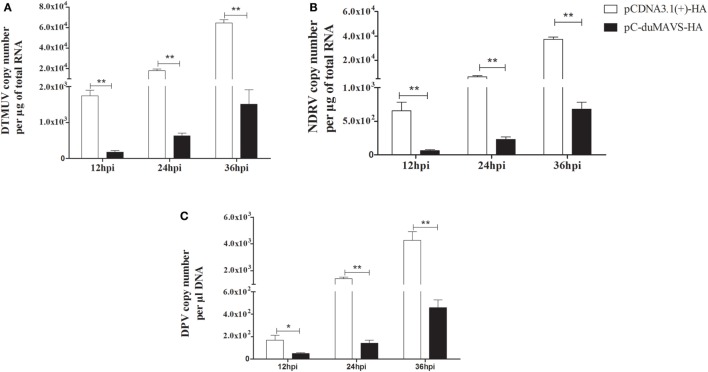
**Antiviral assays of duMAVS in DEFs**. The empty vector and pC-duMAVS-HA were transfected into DEFs. At 24 hpt, the cells were infected with different viruses at 100 TCID_50_ **(A)** DTMUV; **(B)** NDRV; **(C)** DPV. The culture supernatants were collected for detecting the viral titers at 12, 24, and 36 hpi using the RT-qPCR methods and data were repeated three times and each time was analyzed in triplicate. Student’s *t* test was performed to evaluate the differences. *Significant difference (*P* < 0.05); **highly significant difference (*P* < 0.01).

duMAVS can mediate the induction of type I IFNs and ISGs, which are important for defense against viral infection. In order to evaluate antiviral ability, the viral titers were detected at different points in times in the DEFs overexpression of duMAVS after several virus infections, namely, DTMUV (Flaviviridae, a positive sense single strand RNA virus), NDRV (Reoviridae, a dsRNA virus) and DPV (Herpesviridae, a double-strand DNA virus). It was found that the titers of each virus in DEFs transfected with duMAVS were lower than those of cells transfected with empty vector. And there were statistically significant differences at the indicated times after all three viruses infection (Figure 6). Measurement of the viral titers showed that overexpression of duMAVS decreased the viral titer 42.66-fold compared to that in control cells at 36 hpi after DTMUV infection (Figure 6A). Similar results were observed in DEFs infected with NDRV and DPV (Figures 6B,C). The data suggested that duMAVS could inhibit the multiple virus proliferation at points early in infection, and its antiviral activity possesses a certain broad spectrum.

### duMAVS Knockdown Reduces the PolyI:C-Stimulated Induction of IFN-β

**Figure 7:**
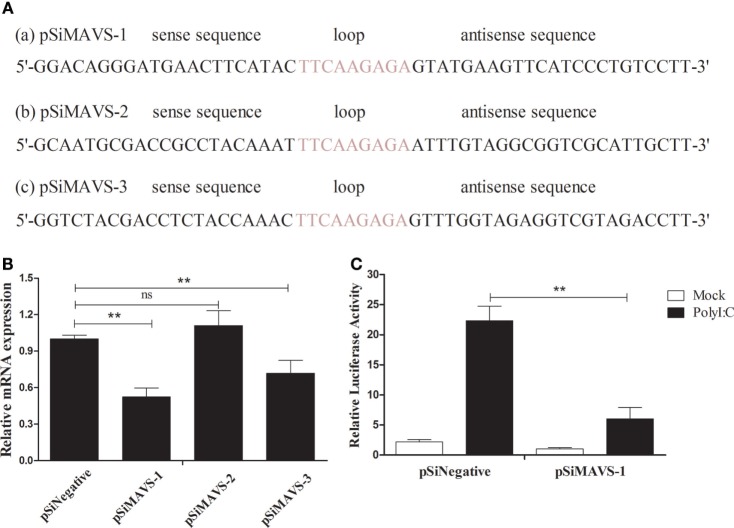
**duMAVS knockdown decreases the polyI:C-stimulated induction of IFN-β**. **(A)** Three siRNA sequences (a, b, and c) targeting the 111–131, 152–173, and 270–290 bp position of duMAVS were prepared, respectively. **(B)** The DEFs were seeded in 6-well plates and transfected with 2.0 μg/well of siRNA. After 20 h of transfection, the cells were collected for the detection of duMAVS expression by RT-qPCR method. The expression of MAVS was first normalized to that of β-actin and then compared to that of the control cells. **(C)** The DEFs were seeded in 24-well plates, cotransfected with pSiMAVS-1 (500 ng/well), IFN-β-luc reporter plasmid (100 ng/well), and pRL-TK plasmid (50 ng/well). After 20 h of transfection, the cells were stimulated with polyI:C (500 ng/well) or remained normal for 12 h, cells were lysed for luciferase assays. Data were means from three independent experiments. Student’s *t*-test was performed to evaluate the differences. *Significant difference (*P* < 0.05); **highly significant difference (*P* < 0.01).

To further explore the function of duMAVS, three siRNA targeting the different position of duMAVS were designed, namely the pSiMAVS-1, pSiMAVS-2, and pSiMAVS-3 (Figure 7A). As shown in Figure 7B, both pSiMAVS-1 and pSiMAVS-3 could decrease the mRNA expression of endogenous duMAVS in DEFs, and the pSiMAVS-1 show stronger interference ability. Therefore, pSiMAVS-1 was selected for the further study. The DEFs were transfected with pSiMAVS-1, pGL3-IFN-β, and pRL-TK plasmids, and then stimulated with polyI:C after 20 h of transfection. As shown in Figure 7C, knockdown of duMAVS resulted in a reduction of IFN-β promoter activity, which indicated that the duMAVS was a very important adaptor for polyI:C-stimulated induction of IFN-β.

### duMAVS Knockdown Reduces Cytokines Production and Antiviral Activity

**Figure 8:**
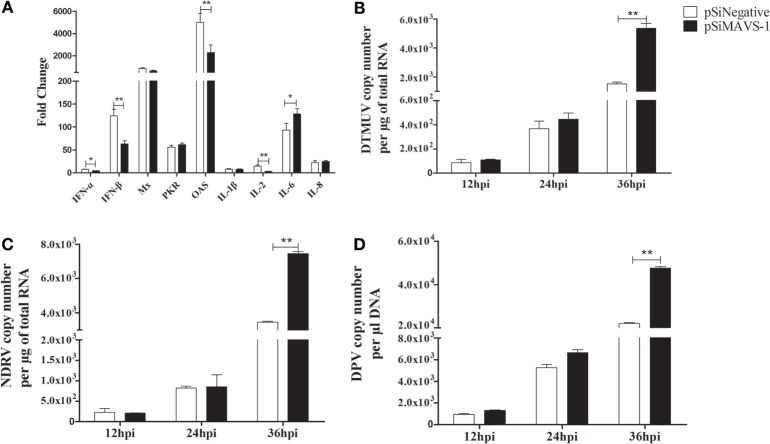
**duMAVS knockdown reduces cytokines production and antiviral activity**. **(A)** The DEFs were seeded in 6-well plates and first transfected with 2 μg/well pSiMAVS-1 or pSiNegative control plasmid. After 20 h transfection, the cells were stimulated with pC-duMDA5-CARD-HA (2 μg/well) for 12 h and then the cells were collected for cytokines detection by RT-qPCR. **(B)** The DEFs were transfected with 2 μg/well pSiMAVS-1 or pSiNegative control plasmid. After 20 h, the cells were infected with DTMUV (100 TCID_50_). The culture supernatants were collected for detecting the viral titers at 12, 24, and 36 hpi by RT-qPCR. The detection of **(C)** NDRV and **(D)** DPV were performed as the same as DTMUV. Data were means from three independent experiments. Student’s *t* test was performed to evaluate the differences. *Significant difference (*P* < 0.05); **highly significant difference (*P* < 0.01).

To investigate the effect of siRNA on cytokines production induced by duMAVS. The DEFs were first transfected with pSiMAVS-1 or pSiNegative control. After 20 h transfection, the cells were stimulated with pC-duMDA5-CARD-HA for 12 h and then the cells were collected for cytokines detection. As shown in Figure 8A, the expression of type I IFNs in siRNA group was significantly lower than that of the control group, similar results were observed in the detection of OAS and IL-2, but the expression of IL-6 was up-regulated in siRNA group. As for other cytokines, there were no significant changes. Additionally, we also detected the antiviral activity of duMAVS knockdown group, it was found that the viral loads (DTMUV, NDRV, and DPV) in the experimental group were higher than those of the control group at 36 hpi (Figures 8B–D). These results suggested that the knockdown of duMAVS could reduce the induction of IFN-α/β and weaken the antiviral activity.

## Discussion

In the current study, Cherry Valley ducks MAVS was cloned and characterized. The duMAVS contains a 1,860 bp ORF and has the characteristic structure domains: a CARD-like region at N-terminal domain, a proline-rich region, and a TM domain at C-terminal domain (Figure 1A). TRAF2, a downstream protein molecule of MAVS, can activate NF-κB and promote antiviral responses mediated by MAVS (8). TRAF2-binding motif was found in the proline-rich domain of duMAVS and highly conserved in ducks, chickens, and geese (Figure 1A). The amino acid sequence of duMAVS shared a 99.2% homology with the predicated sequence of duMAVS using the Clustal W method, which was higher than 97.9% of the others (29–31). Multiple sequence alignments indicated that the amino acid sequence of duMAVS only exhibited 22.0% identity to human, 58.4% to chicken, and 77.7% to goose. However, the CARD-like domain homology between ducks and other species was higher as compared to their full length homology, which indicated that CARD remained highly conserved during evolution. Notably, the homology between duck and goose was highest, followed by chicken (Table 2). Similar results were also observed in the phylogenetic tree (Figure 1B). These results revealed that duMAVS had a close genetic relationship to other birds, especially the goose and chicken.

Since MAVS is an essential protein of innate immunity, study of its tissue distribution will contribute to a better understanding of its function. In this study, although duMAVS was expressed in all tissues tested with a broad expression spectrum, the highest expression was found in the pancreas, followed by the liver, heart, and lung, there was a relative low expression in the spleen and bursa of Fabricius. The result of duMAVS tissue distribution was not identical with others (29). The development of tissues and organs from different aged ducks was in different stages, which may explain the different tissue distribution. The detailed biological function of this tissue distribution of duMAVS will require a further study. In animal experiments, DTMUV was used to infect the 21-day-old ducks. It was found that the level of duMAVS was significantly upregulated in the spleen and brain in DTMUV-infected ducks, particularly in the brains (Figure 3), similar results were observed in the brain and spleen of DPV-infected ducks (30). However, the expression of duMAVS was downregulated in the spleen of NDRV-infected ducks (31). These results indicated that duMAVS-dependent pathway might be involved in the antiviral immune response. Actually, other studies have shown that MAVS was essential for the establishment of immune response to West Nile virus infection, which also a member of Flaviviridae (32).

It is well known that MAVS-dependent signal pathway can activate IFN-β through NF-κB or IRFs (5, 12). In the current study, dual-luciferase reporter gene assay was performed to detect the luciferase activity of various plasmids. The results of overexpression and RNA interference showed that duMAVS full length could significantly activate IFN-β luciferase activity as compared to empty vector, but the luciferase activity of IFN-β was not significantly enhanced in duMAVS-CARD- and duMAVS-dCARD-transfected DEFs (Figure 4B). Similar results were found in the detection of luciferase activity of NF-κB and IRF7 (Figures 4C,D), indicating that CARD and dCARD domains were necessary for this activation. Previous studies also demonstrated that CARD and TM domain of MAVS were necessary for the induction of IFN-β, and TM domain could target MAVS to the mitochondrial membrane, and exact localization of MAVS was essential for its function in signal pathway (5, 12). Taken together, duMAVS could significantly activate the IFN-β luciferase activity by NF-κB and IRF7, but duMAVS-CARD and dCARD domains are both required for this function.

In other studies, the expression levels of IFN-I and ISGs were induced by the overexpression of MAVS (14, 15). While several cytokines and ISGs were analyzed by RT-qPCR in duMAVS-overexpressing DEFs, most were upregulated, including the IFN-β, Mx, OAS, IL-2, IL-6, and IL-8 (Figure 5). Moreover, the expressions of IFN-β, OAS, and IL-2 were downregulated in duMAVS knockdown group (Figure 8A), which indicating that duMAVS played an important role in regulating the induction of cytokines, especially the IFN-β. A large number of IFNs and ISGs can suppress viral replication. Furthermore, it has been reported that MAVS played an important role in defending against the RNA and DNA viruses (33). In order to investigate whether the duMAVS had a broad-spectrum antiviral function, the different types of viruses (DTMUV, NDRV, and DPV) were used to detect the antiviral activity of duMAVS by the overexpression and knockdown methods. Our results showed that the viral titers in DEFs transfected with duMAVS full length were lower than those of DEFs transfected with empty vector (Figure 6), however, the viral titers in duMAVS knockdown DEFs were higher than those of control cells at 36hpi (Figure 8). Recently, MAVS has been necessary for controlling the replication and spread of many viruses, such as West Nile virus, rotavirus, and herpes simplex virus (32, 34, 35). These results indicated that antiviral activity of duMAVS had a certain universality.

In conclusion, Cherry Valley duck MAVS was demonstrated, and its function was analyzed in this study. The expression of duMAVS was very extensive. duMAVS was involved in immune response to the virus infection *in vivo*. Many downstream factors, including IFN-β, Mx, and OAS, could be induced by duMAVS. Furthermore, duMAVS could suppress the replication of various viruses *in vitro*. These results would contribute to better understanding the innate immune system of ducks.

## Author Contributions

NL and TH performed the main experiment, analyzed data, and wrote the manuscript. RL, YW, MG, ZC, YC, and SL performed the experiment and wrote the discussion. LW and TC designed the experiment and reviewed the manuscript.

## Conflict of Interest Statement

The authors declare that the research was conducted in the absence of any commercial or financial relationships that could be construed as a potential conflict of interest.

## Footnotes

^1^http://www.ncbi.nlm.nih.gov/blast

^2^http://ch.embnet.org/software/BOX_form.html

^3^http://smart.embl-heidelberg.de/
